# Functional and Structural Plasticity of Brain in Elite Karate Athletes

**DOI:** 10.1155/2018/8310975

**Published:** 2018-10-23

**Authors:** Adil Deniz Duru, Taylan Hayri Balcioglu

**Affiliations:** Faculty of Sports Science, Marmara University, Istanbul, Turkey

## Abstract

The structural and functional neural differences between the elite karate athletes and control group have been investigated in the concept of this study. 13 elite karate athletes and age-gender matched 13 volunteers who have not performed regular exercises participated in the study. Magnetic resonance imaging was used to acquire the anatomical and functional maps. T1-weighted anatomical images were segmented to form gray and white matter images. Voxel-based morphometry is used to elucidate the differences between the groups. Moreover, resting state functional measurements had been done, and group independent component analysis was implemented in order to exhibit the resting state networks. Then, second-level general linear models were used to compute the statistical maps. It has been revealed that increased GM volume values of inferior/superior temporal, occipital, premotor cortex, and temporal pole superior were present for the elite athletes. Additionally, WM values were found to be increased in caudate nucleus, hypothalamus, and mammilary region for the elite karate players. Similarly, for the elite karate players, the brain regions involved in the movement planning and visual perception are found to have higher connectivity values. The differences in these findings can be thought to be originated from the advances gained through the several years of training which is required to be an elite karate athlete.

## 1. Introduction

Sports performance seems to be directly related to the advances gained throughout the life. The performance differences observed between athletes who have similar physical fitness levels can be explained with the structural and functional plasticity occurred in their brain tissue. In the last decade, with the advancements in the brain imaging technology, neuroplasticity that occurred due to motor learning or training has been investigated in a large number of studies. For instance, it has been shown that cortical thickness of motor area increases after a complex balance task that lasts for an hour [1]. Nevertheless, most of the studies aimed to identify the changes caused by long-term motor training. For handball players, the volume of gray matter (GM) was found to be increased in hand control areas while for ballet dancers, a similar result was obtained in foot areas [2]. And yet, contrary findings as decrements of GM volume in motor cortex and superior frontal gyrus have been reported by Hanggi et al., where elite dancers were compared with nondancers [3]. Besides this, variations in neural matter density have been observed in segmented images of motor cortex due to motor skill learning [4, 5, 6]. For instance, voxel-based morphometry (VBM) analysis exhibited gray matter density increments in right and medial cerebellar areas for badminton players [4] as well as in thalamus and precentral gyrus for divers [7]. Moreover, white matter (WM) density changes in frontal and in parietal lobes were shown to be related with the motor learning [5, 8]. As an index of white matter density, decreased fractional anisotropy (FA) values of black belt karate players in superior cerebellar peduncles and primary motor cortex were exhibited when compared with the control subjects [9].

In addition to structural changes, motor skill learning has an effect on the functioning of the brain [10]. It can be thought that the changes of the underlying structure should have an effect on the functionality. To investigate this idea, among the functional brain imaging modalities, with a superior spatial resolution, as a noninvasive technique, fMRI has been widely used to monitor the dynamics of the brain by means of blood oxygenation. Resting state measurements are seen as the most suitable technique to determine the differences that are caused by the gained experience or learning. Due to its nature, resting state measurements do not require any motor movement or performing a cognitive task. Among the RSNs, alterations in frontoparietal and cerebellar networks were shown to be caused by motor learning [11].

To become an elite athlete, several hours of training is one of the requirements that is needed to be accomplished. It has been shown that changes can occur in brain tissue with respect to repetitive performance of the motor exercise lasting in years [12]. Moreover, resting state functional responses of the brain can vary even after a single aerobic exercise session [13].

Up to our knowledge, morphometric differences between elite karate players and control subjects have not been investigated by means of gray matter distribution. In the concept of this study, it is aimed to determine the morphometric differences as well as the resting state networks differences between elite karate players and control subjects.

## 2. Methods

### 2.1. Participants

Twenty-six subjects participated in the study (13 elite karate athletes and 13 control group volunteers). Elite karate athlete group members (6 females and 7 males) have an average age of 22.3 (*σ*=4.6), while it was 26.7 (*σ*=5.8) for age and gender matched healthy control subjects. Elite subject group members reported that they had practiced karate sports at least 10 years, and they are performing at least fifteen hours' training program weekly. Each elite karate player had attended the European or World Karate championships and achieved more than three medals. Karate athletes hold the karate black belt for 8 years on average. On the other hand, control group subjects had not experienced regular sports training programs.

Subjects suffering from a neurological disorder were excluded from the study. Data were acquired at the MR Imaging center of Hulusi Behcet Life Sciences Center, Istanbul University, Istanbul Medical School using a 3*T* Philips Achieva Scanner. The measurements were conducted in accordance with the declaration of Helsinki. The study was approved by Marmara University Health Institute Review Board with the document number 09.2016.414(15.07.2016). The subjects have been informed about the MR measurements, and written informed consent was read and signed by all participants prior to measurements.

### 2.2. Anatomical and Resting State fMRI

By the advanced technology, anatomical structure of the brain tissue can be characterized in a volume having less than one millimeter cube size. The volumetric distribution of the gray and white matter tissue exhibits the neuroplasticity occurred in localized regions. T1-weighted MR images are obtained with a slice thickness of 1 mm and 0.97 mm resolution in the *xy* plane. 180 slices are formed for each volume (TR 8.74 ms, TE 3.9 ms).

Vascular and metabolic responses increase with respect to a stimulus or during the implementation of a task. However, in the absence of a task or stimulus, there is still a demand of resources for internal processing. fMRI, as a noninvasive neural activity imaging technique, uses variation of the deoxyhemoglobin in venous blood in order to monitor the blood oxygenation level dependent (BOLD) contrast. In this study, functional images were acquired using a gradient echo, echo planar imaging (EPI) sequence for a duration of 7 minutes in eyes closed condition (transverse orientation, TR 2 s, TE 30 ms, 77° flip angle, 36 slices, and 4.0 mm slice thickness with a voxel size of 1.86 mm in *x* and *y* space).

### 2.3. Image Processing Pipeline

In this study, VBM was used to exert the possible anatomical differences of the brain between groups of subjects in the voxel level [14]. VBM requires the input images to be in the same spatial space. Thus, spatial normalization of the anatomical images is required in order to compare the groups of subjects. Normalization procedure is used to transform all of the subjects' images to a common template [15, 16]. The average template image of the Montreal Neurologic Institute (MNI 152) has been used as the reference template [17]. Afterwards, normalized T1-weighted anatomical images were segmented into gray matter (GM), white matter (WM), and cerebrospinal fluid (CSF) tissues. Segmentation and normalization procedure were carried out using Diffeomorphic Anatomical Registration Through Exponentiated Lie Algebra (DARTEL) [6, 18]. After the implementation of preprocessing steps, a general linear model was used to form the statistical parameter maps. Age of the subjects, their intracranial volume, and gender information were used as covariates in the design of the GLM, both for GM and WM tissues, separately. All of these analyses had been carried out using statistical parametric mapping (SPM8) software [19]. After the implementation of the GLM, since a huge amount of statistical tests were being computed, multiple comparison correction was performed. Family-wise error (FWE) correction was performed using *p* < 0.05 with an extend threshold of 20 voxels. The GM and WM differences deduced with respect to elite > control contrast are given in Tables 1 and 2, respectively.

After the anatomical measurements, resting state functional images were obtained. During fMRI measurements, in each time instance, a 2d image has been reconstructed while the whole brain consists of several of these maps. The state-of-the-art approach to the preprocessing of the functional images consists of slice timing, realignment, normalization, and smoothing operations [20]. Slice timing process was used to correct the order of the fMRI acquisition to avoid timing mismatches [21]. Then, realignment process was applied for motion correction by coregistering each 3d image to a reference volume. Spatial normalization procedure was applied to warp each subject's brain into a common reference space (MNI 152). This procedure enables us to compare the changes observed between groups of subjects.

Finally, the normalized volumes were smoothed to increase the signal-to-noise ratio. After the smoothing operation, each voxel had the weighted average values of the neighbouring voxels based on a Gaussian function. The smoothed 3d images were further used to determine the independent components which were assigned as the intrinsic connectivity networks. Spatial independent component analysis has been performed in order to identify several spatial components with their time courses using GIFT toolbox developed by Calhoun et al [22]. fMRI was performed during resting condition for seven minutes. Thus, the minimum frequency content that can be observed in the BOLD time series is less than 0.005 Hz. Low-frequency oscillation in the 0.01 to 0.08 Hz range was thought to be the source of RSNs.

Each component deduced by the GIFT toolbox was checked with respect to findings reported in the literature. As a result, somatomotor, dorsal attention, visual, and default mode network (DMN) had been identified. The network maps of each subject were back reconstructed, and *t*-test statistics were applied for each network map set in order to exhibit the differences between the elite and control group. Second-level GLMs were used to implement the statistics using the subjects' intracranial volume, age, and gender values as regressors. SPM8 was used to perform the GLM analysis.

## 3. Results

### 3.1. Voxel-Based Morphometry Differences

There were no significant differences of whole GM and WM volumes between groups. The mean volume of GM for the elite group was 737.02(*σ*=62.79)  cm^3^ while it was 708.78(*σ*=58.92)  cm^3^ for the control group (*p*=0.249). The elite group had an average of 514.74(*σ*=51.42)  cm^3^ WM while the control group had 487.71(*σ*=41.44)  cm^3^(*p*=0.191). GM and WM volume values of all participants are summarized in Table 3.

**Figure 1:**
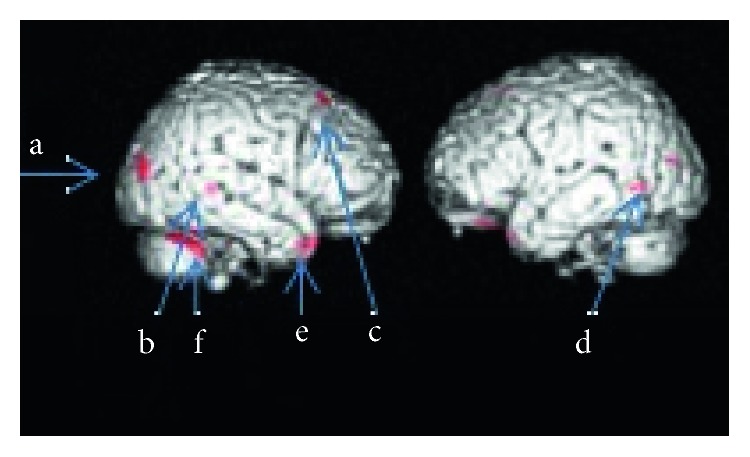
Significant GM differences are (elite > control) visualized with red blobs on the structural template cortical surface.

Parametric statistical tests have been performed in a voxel-wise manner while multiple comparisons were corrected based on random field theory. Extend threshold was set as 20 voxels, and FWE-corrected results are demonstrated for the GM differences between elite and control subject groups (Figure 1 and Table 1). Compared with the control group, the elite group had increased GM volume in right (R) inferior temporal sulcus, premotor cortex (R), temporal pole superior (R), cerebellum, and lateral occipital sulcus.

**Figure 2:**
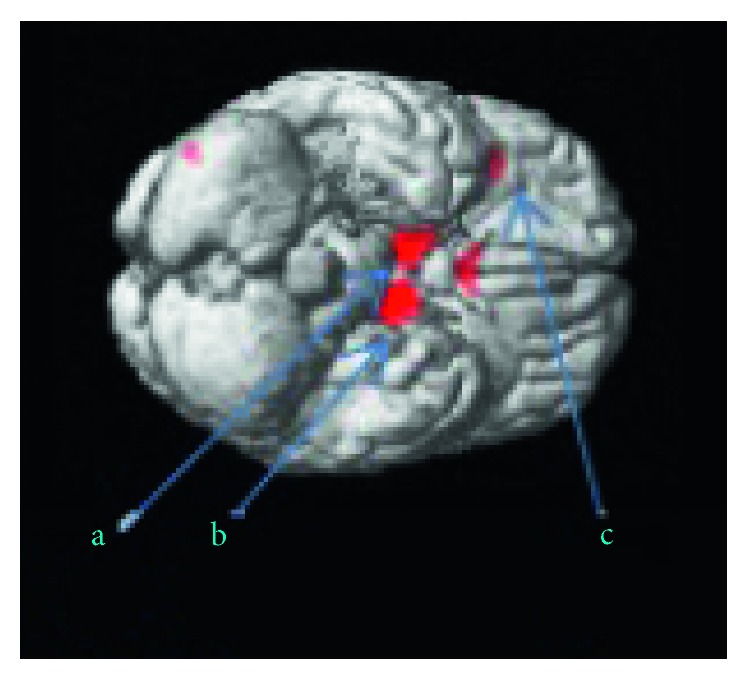
Significant WM differences are (elite > control) visualized with red blobs on the structural template cortical surface.

On the other hand, WM increments have been obtained in caudate nucleus, left insula, mammillary region, and hypothalamus for the elite karate athletes as summarized in Figure 2 and Table 2.

### 3.2. Functional Results

**Figure 3:**
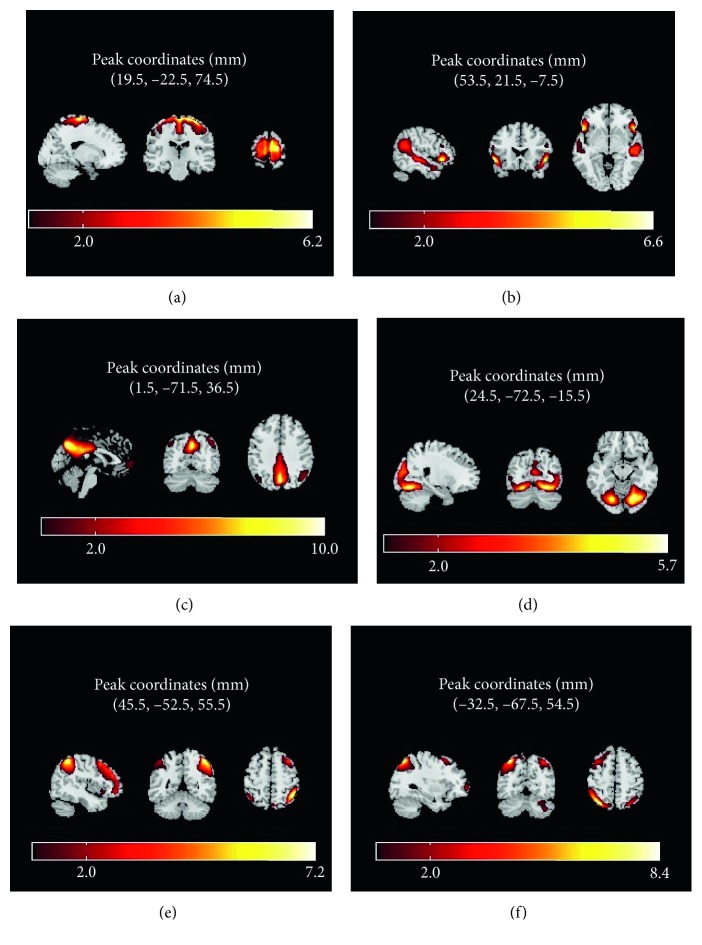
Mean RSN maps of the elite athletes and control subjects thresholded by *Z* > 2. Somatomotor network (a), executive (b), default mode network (c), visual (d), frontoparietal network, and (e) and (f).

Resting state functional images are used to obtain intrinsic connectivity networks by the use of group independent component analysis. The mean spatial distribution of the auditory, somatomotor, visual, DMN, and dorsal attention networks over subjects are shown in Figure 3 using threshold *Z* > 2. Remaining components have not been taken into consideration for further analysis since these were mostly artefacted components.

For each functional network, the differences between groups are investigated by the use of a second-level SPM analysis. Error probability was set to *p* < 0.001 uncorrected for multiple comparisons since none of the voxels survived when FWE correction was imposed with *p* < 0.05.

Cuneus area as a part of visual network was observed to be more activated for the karate players when compared with the control subjects. In addition to this, medial frontal gyrus and superior temporal gyrus which are known to be part of the DMN were shown to have higher activation values for the karate players.

Increased intrinsic activity in postcentral gyrus, superior frontal gyrus, left inferior frontal gyrus, and cerebellum has been observed for elite karate players as summarized in Table 4.

The significant findings of the present study were reported using the contrast elite players > control subjects. When the contrast of control subjects > elite players was used, none of the brain areas was found to have significant greater intrinsic activation pattern.

## 4. Discussion

### 4.1. Anatomical Differences between Elite and Control

Existing literature points out that right inferior temporal and occipital cortex GM volume values decrease in professional video game players and in the patients suffering from addiction of online games when compared with healthy controls [23]. This volume loss might be related with the long-term exposure of harmful visual stimuli received from the online computer games. The inferior temporal cortex acts as a gateway for visual perception and memory [24]. In the concept of this study, unlike to the effects of the harmful visual stimuli presentation, increased gray matter volume had been deduced for elite karate players in the right inferior temporal area which is a part of the visual movement perception. It is thought that the neuroplasticity of this area can be formed via motor learning [25].

If a subject is classified as an elite karate athlete, he or she should have higher motor coordination capacity and should perform training for a long term. Since we do not have the brain images of the elite karate athletes prior to sports training, it is not possible to conclude that the differences observed in the brain anatomy is just affected by the long-term training period. However, it has been shown that aerobic capacity is a major component to achieve high karate performance [26]. Thus, GM increments in the premotor cortex of the elite karate players can be thought to be partly originated from the advances gained through the several years of training. Nevertheless, GM increments of the premotor cortex were observed in the period of a whole body balancing task training parallel to the improvement of the performance [7]. For all trained athletes, premotor cortex gray matter increments should be observed since the most of the physical exercises require the control of body orientation.

Moreover, it has been shown that the subjects having lesions in premotor cortex and supplementary motor area experienced difficulties when they need to recall movements from their memory as a response to a given stimulus in the form of a sensory cue. Based on these findings, it was thought that sensory conditional motor performance might be related with the premotor cortex and supplementary motor area [27]. Since the karate exercises include the implementation of complex movements that consist of several types of visual stimulation coming from the opponent, it is not an unexpected issue to have premotor gray matter volume increments in the elite karate group when compared with the controls in the concept of our study.

In this study, we found that gray matter density of right temporal pole superior (RTPS) in elite karate players' brain regions was higher than others. In the literature, a VBM study reported the relationship between the GM volume of right temporal pole and perspective taking process [28]. Moreover, the superior temporal pole was shown to have a role in the representation of the social concepts [29].

A person who is trained or skilled in exercises, or learned art, may have an improved capacity of understanding the cognitive and affective profile of the other people [30]. Our finding of the increased GM in RTPS can be associated with the above definition by means of perspective taking.

Visually guided motor tasks activate premotor and inferior parietal cortex [31]. The activity pattern starting from primary visual cortex and passing through ventral surface into the temporal cortex is known as the ventral stream while dorsal stream connects the occipital pattern to parietal cortex through the dorsal surface [32]. Ventral stream is known to be responsible from the perception of visual shapes of the objects while the dorsal stream is thought to be related with the temporally tracking of their spatial location. When we identify a motion process, activity of visual motion area (V5, MT) arises [33]. In our study, the increments of GM in inferior and superior temporal gyrus and premotor cortex might be related with several hours of exercise time spent throughout the life in elite karate players. These exercise sessions consist of phases where an athlete has to predict the movements of his rival.

Dorsal prefrontal cortex plays an important role in the selection and representation of actions, and it projects to cerebellum [34]. In our study, GM density in the cerebellum of elite karate players was found to be larger than the others. In the literature, cerebellum is stated as one of the specialized regions which is responsible for the implementation of learned or automatic movements [35].

In addition to GM differences, in our study, caudate nucleus, left insula, mammillary region, and hypothalamus white matter density values were found to be increased for the elite karate athletes. These regions can be thought to include more myelinated axons that affect the speed of the neural signalling [36]. These findings should be further supported by the DTI measurements to ensure the differences of white matter distribution. In a DTI study, FA values of uncinate fasciculus and cingulum were found to be positively correlated with the aerobic fitness level of the subjects [37]. Moreover, Roberts et al. showed the association between the parameters as motor coordination, the time length of expertise, the age that the karate players started training, and white matter integrity in the cerebellum [8].

### 4.2. Functional Differences between Elite and Control

RSNs exhibited in this study are compared between two groups of subjects in order to elucidate the functional differences that is thought to be based on the neuroplasticity due to the implementation of long-term exercises. In instance, effects of running were shown as improved performance in cognitive tasks that is thought to be originated from the neuroplasticity in prefrontal cortex [38]. In a recent study, an evidence of the positive influences of short-term exercises (12 weeks) on the cognitive task performance has been associated with the increased functional connectivity during resting state especially in the prefrontal cortex and hippocampus [39]. In the concept of our study, these areas were found to have greater connectivity values which are in agreement with the current literature.

In the concept of this study, superior temporal area (posterior parietal cortex, PPC) activation was found to be greater than the corresponding values of the control group. In the literature, PPC and precuneus have been reported to have a role in visual perception, planning as well as execution of reach [40] which are the major features that elite karate players should have.

In the executive control network, cerebellum crus1 was found to have a greater functional connectivity value in karate players. Cerebellum and sensorimotor cortex have a role in the organization of the movements. Additionally, postcentral gyrus is involved in the sensorimotor processing [41].

## 5. Conclusion

It can be concluded that the GM superiority can be an underlying cause of the differences in RSNs. Moreover, GM differences can be a basis of the neural efficiency gained by the regular long-term karate exercises. However, the WM differences should be analyzed by the use of DTI measurements. Up to our knowledge, this is the first study that investigates the functional neural dynamics using resting state fMRI on elite karate athletes.

The major drawback of this study is the limited number of elite karate athletes. The limited number of karate players is due to the fact that we required difficulty to reach conditions for the definition of elite karate players. It is not an easy process for a karate player to achieve the required conditions even when he holds a black belt.

## Figures and Tables

**Table 1:** Voxel-based differences of GM deduced by using the contrast elite > control subjects exhibiting six significant clusters having at least 20 clusters are summarized.

Cluster p (FWE)	Cluster k	Peak p (FWE)	Peak T	*x* (mm)	*y* (mm)	*z* (mm)	Label
0.0001	215	0.0002	9.89	58.5	−52.5	−27	b (inferior temporal, R)
		0.0355	6.52	55.5	−48	−40.5	e (temporal pole superior, R)
0.0088	29	0.0008	8.77	28	20	58	c (premotor cortex, R)
0.0000	242	0.0012	8.56	18	19.5	−31.5	f (cerebellum, R)
		0.0014	8.42	34.5	39	−24	
		0.0017	8.33	33	31.5	−27	
0.0041	50	0.0024	8.11	46.5	−85.5	15	a (occipital-temporal v5 mi, R)
0.0118	22	0.0042	7.76	46.5	−43.5	1.5	b (Inferior/superior temporal gyrus, ba20, R)
0.0118	22	0.0083	7.36	−64.5	−61.5	−3	d (superior temporal, L)

Each cluster is represented with a letter in Figure 1. Cluster level FWE corrected probability values are reported with the MNI coordinates with the statistical values of the peak voxel in the cluster. Peak T value stands for the maximum of *t* statistic value for a voxel in a cluster. Peak p (FWE) stands for the voxel level significance value, where at least one cluster with unspecified number of voxels is above the threshold (*p* < 0.05). The number of voxels above the threshold in a cluster is denoted with cluster *k* while the Cluster p (FWE) is the cluster level probability. MNI coordinates and corresponding anatomical names are represented by *x*, *y*, *z* and label, variables, respectively.

**Table 2:** Voxel-based differences of WM deduced by using the contrast elite > control subjects exhibiting four significant clusters having at least 20 clusters are summarized.

Cluster p (FWE)	Cluster Equivk	Peak p (FWE)	Peak T	*x* (mm)	*y* (mm)	*z* (mm)	Label
0.0002	198	0.0000	10.39	−9	−7.5	−19.5	Mammillary region (b)
0.0006	141	0.0005	8.76	10.5	−4.5	−16.5	Hypothalamus (a)
		0.0156	6.70	15	3	−13.5	Hypothalamus (a)
0.0017	97	0.0023	7.82	6	16.5	7.5	Caudate nucleus right
0.0097	33	0.0064	7.21	−37.5	25.5	4.5	Left insula (c)

Each cluster is represented with a letter. The location of the clusters can be seen in Figure 2. Cluster level FWE corrected probability values are reported with the MNI coordinates with the statistical values of the peak voxel in the cluster. Peak T value stands for the maximum of t statistic value for a voxel in a cluster. Peak p (FWE) stands for the voxel level significance value where at least one cluster with unspecified number of voxels is above the threshold (*p* < 0.05). The number of voxels above the threshold in a cluster is denoted with the cluster k while the cluster p (FWE) is the cluster level probability. MNI coordinates and corresponding anatomical names are represented by *x*, *y*, *z* and label, variables, respectively.

**Table 3:** Gray matter (GM) and white matter (WM) volume values (cm^3^) of each participant.

Gender	Participant	GM volume (cm^3^)	WM volume (cm^3^)
Male	Elite 1	773.48	535.71
Male	Elite 2	843.61	580.24
Male	Elite 3	714.81	497.80
Male	Elite 4	764.12	531.58
Male	Elite 5	809.33	580.41
Male	Elite 6	628.99	424.86
Male	Elite 7	757.61	523.80
Female	Elite 8	690.93	474.24
Female	Elite 9	806.75	602.38
Female	Elite 10	712.94	482.64
Female	Elite 11	719.62	506.68
Female	Elite 12	707.83	491.33
Female	Elite 13	651.28	460.01
Male	Control 1	778.48	548.15
Male	Control 2	809.75	551.04
Male	Control 3	685.72	512.70
Male	Control 4	720.53	487.86
Male	Control 5	710.02	478.28
Male	Control 6	755.10	531.94
Male	Control 7	787.03	514.16
Female	Control 8	651.68	439.27
Female	Control 9	684.17	452.25
Female	Control 10	647.41	458.17
Female	Control 11	645.99	453.02
Female	Control 12	707.55	488.06
Female	Control 13	630.77	425.28

**Table 4:** Significant differences between elite karate players and control group deduced by the analysis of the second-level GLM for each RSN map.

T	Z	p (unc)	*x*	*y*	*z*	Label
3.72	3.22	<0.001	4	−88	6	Cuneus BA18 (R)
5.21	4.13	<0.0001	2	56	−6	Medial frontal gyrus (R)
5.00	4.01	<0.0001	60	−60	26	Superior temporal gyrus (R)
4.66	3.82	<0.0001	24	−34	72	Postcentral gyrus (R)
3.74	3.24	<0.001	44	−34	58	
3.60	3.14	<0.001	22	−4	66	Superior frontal gyrus (R)
4.51	3.73	<0.0001	4	26	50	Superior frontal gyrus, frontal sup medial (R)
4.22	3.55	<0.001	−42	32	−2	Left inferior frontal gyrus (L)
4.15	3.49	<0.001	10	−76	−28	Cerebellum crus1 (R)

## Data Availability

The data used to support the findings of this study are available from the corresponding author upon request.
